# Long sleep duration and good sleep quality reduced incident peptic ulcer disease in a large Taiwanese population follow-up study

**DOI:** 10.7150/ijms.103639

**Published:** 2025-01-21

**Authors:** Yi-Hsiang Huang, Hsin-Yi Huang, Jia-In Lee, Ting-Yi Wu, Shu-Pin Huang, Jiun-Hung Geng, Chao-Hung Kuo, Szu-Chia Chen

**Affiliations:** 1 Department of Internal Medicine, Kaohsiung Medical University Hospital, Kaohsiung Medical University, Kaohsiung, Taiwan.; 2 Teaching and Research Center, Kaohsiung Municipal Siaogang Hospital, Kaohsiung Medical University, Kaohsiung, Taiwan.; 3 Department of Psychiatry, Kaohsiung Medical University Hospital, Kaohsiung Medical University, Kaohsiung, Taiwan.; 4 Department of Urology, Kaohsiung Medical University Hospital, Kaohsiung Medical University, Kaohsiung, Taiwan.; 5 Department of Urology, Kaohsiung Municipal Siaogang Hospital, Kaohsiung Medical University Hospital, Kaohsiung Medical University, Kaohsiung, Taiwan.; 6 School of Medicine, College of Medicine, Kaohsiung Medical University, Kaohsiung 807, Taiwan.; 7 Department of Internal Medicine, Kaohsiung Municipal Siaogang Hospital, Kaohsiung Medical University Hospital, Kaohsiung Medical University, Kaohsiung, Taiwan.; 8 Division of Gastroenterology, Department of Internal Medicine, Kaohsiung Medical University Hospital, Kaohsiung Medical University, Kaohsiung, Taiwan.; 9 Division of Nephrology, Department of Internal Medicine, Kaohsiung Medical University Hospital, Kaohsiung Medical University, Kaohsiung, Taiwan.

**Keywords:** sleep duration, sleep quality, peptic ulcer disease, Taiwan biobank

## Abstract

Poor sleep has been associated with diseases including cardiovascular, obesity and mental disorders. However, there is limited information on the correlation between sleep duration and quality with peptic ulcer disease (PUD). This study aimed to investigate the impact of sleep duration and quality on the incidence of PUD in a large Taiwanese population follow-up study. The study participants were recruited from the Taiwan Biobank. Sleep duration, sleep quality, and the presence of PUD were assessed using self-reported questionnaires. The participants were categorized into three groups based on sleep duration: < 7 hours/day, 7 hours/day, and > 7 hours/day. Sleep quality was divided into five levels: very poor, poor, normal, good and very good. The association between sleep duration and quality with incident PUD was analyzed using multiple logistic regression after controlling for confounders. We collected data from 22,561 participants (excluding those with pre-existing PUD, missing basic information, or lacking sleep data). Over an average follow-up period of 43 months, multivariable analysis showed that sleep duration > 7 hours/day (*vs.* < 7 hours/day; hazard ratio [HR], 0.771; 95% confidence interval [CI], 0.668 to 0.890; *p* < 0.001) was significantly associated with incident PUD. Further, sleep duration (per 1 hour/day; HR, 0.933; 95% CI, 0.889 to 0.979; *p* = 0.005) was also significantly associated with incident PUD. Those with poor sleep quality (*vs.* very poor quality; HR, 0.649; 95% CI, 0.491 to 0.858; *p* = 0.002), normal sleep quality (*vs.* very poor quality; HR, 0.611; 95% CI, 0.469 to 0.795; *p* < 0.001), good sleep quality (*vs.* very poor quality; HR, 0.507; 95% CI, 0.382 to 0.671; *p* < 0.001), and very good sleep quality (*vs.* very poor quality; HR, 0.493; 95% CI, 0.367 to 0.662; *p* < 0.001) were significantly associated with incident PUD. We found that longer sleep duration and better sleep quality were independent protective factors for PUD. Future research should explore the underlying mechanisms and verify whether improving sleep can directly reduce the incidence of PUD.

## Introduction

Both sleep duration and quality play crucial roles in maintaining overall health and well-being 1. Regarding sleep duration, the American Academy of Sleep Medicine and the Sleep Research Society recommend 7 hours or more per night for adults to promote optimal health 2-4. Sleep quality is determined by the frequency of awakenings during the night, along with the percentage, duration, and type of sleep stages 5, 6. According to the National Sleep Foundation, shorter sleep latencies, fewer awakenings, reduced wake after sleep onset, and higher sleep efficiency are indicators of good sleep quality 7. A short duration of sleep has been associated with various adverse health outcomes, including obesity, hypertension, type 2 diabetes, metabolic syndrome and cardiovascular diseases 8-12. In contrast, a prolonged sleep duration has been associated with increased risks of cerebrovascular events, coronary artery heart disease, and diabetes 13-15. A U-shaped association between overall mortality and sleep duration has been suggested, with the lowest risk at 7 hours of sleep; however, the results of previous studies have been conflicting 2, 3. Poor sleep quality has been reported to lead to neuropsychological problems, including anxiety, depression, and cognitive impairment 1, 16.

Peptic ulcer disease (PUD) is a common gastrointestinal disease involving mucosal break in the stomach or proximal duodenum 17. Helicobacter pylori (H. pylori) infection and the use of nonsteroidal anti-inflammatory drugs (NSAIDs) are the most common etiologies of PUD 17-19. The global incidence of PUD has been decreasing over the past decades (from 63.84 in 1990 to 44.26 per 100,000 population in 2019), possibly due to H. pylori eradication therapy, effective gastric acid-suppressing medications, and awareness of the adverse effects of NSAIDs 20-22. The hospitalization rate of PUD varies among countries, with the highest incidence in Eastern Europe and Eastern Asia 20. Despite the decreasing trends in incidence and mortality rates, PUD and associated complications of bleeding and perforation remain a major burden on healthcare systems worldwide, particularly as hospitalized patients tend to be older, with multiple comorbidities, or taking anticoagulants and NSAIDs 23, 24.

Sleep disturbance has also been linked to gastrointestinal diseases 25, and previous studies have reported associations between poor sleep quality with PUD recurrence and rebleeding 26-28. A Korean study reported that women who slept less than 7 hours a night had nearly twice the risk of having PUD compared to those who slept more than 9 hours a night 29. However, the cross-sectional retrospective design of the study limited the validity of the association. Due to the limited data and lack of large retrospective cohort studies addressing this issue, the present study aimed to investigate the associations between sleep duration and quality with the incidence of PUD using longitudinal data from 22,561 participants in the Taiwan Biobank (TWB).

## Materials and Methods

### Data source and study population

This study was based on data from the TWB, a nationwide prospective community-based research database. Participants aged 30 to 70 years without cancer have been enrolled in the TWB from over 30 recruitment centers in Taiwan since 2008. The methods and details regarding the establishment of the TWB have been described previously. Each participant underwent a health interview, a general physical examination, and biochemical tests of blood and urine. Data on lifestyle practices, dietary habits, environmental exposures, genomic characteristics, and long-term health outcomes were collected. Ethical approval for TWB was granted by the Ethics and Governance Council of the TWB and the Institutional Review Board on Biomedical Science Research, Academia Sinica, Taiwan.

**Figure 1:**
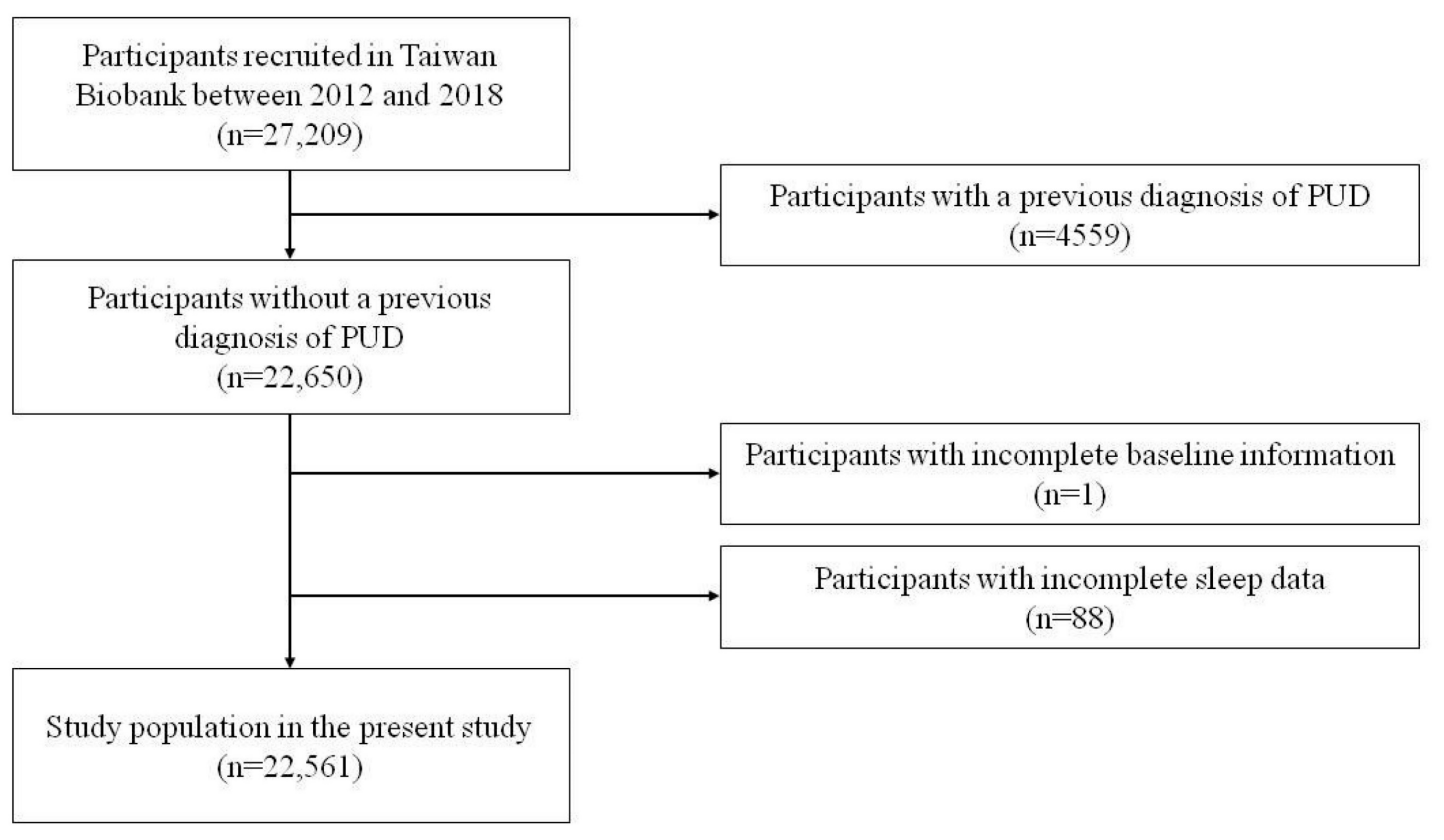
Flowchart of study population (PUD = Peptic ulcer disease).

A total of 27,209 participants with long-term follow-up data were enrolled in the study. Initially, the participants were screened for PUD, and those with known PUD were excluded (*n* = 4559). Participants with missing baseline information (*n* = 1) and those with incomplete sleep data (*n* = 88) were also excluded, and the remaining 22,561 participants were enrolled (Figure 1). The mean follow-up period was 43 months. Written informed consent was obtained from all included participants. This study was conducted according to the Declaration of Helsinki and approved by the Institutional Review Board of Kaohsiung Medical University Hospital (KMUHIRB-E(I)-20210058).

Baseline data including age, sex, body mass index (BMI), smoking status, alcohol status, physical activity, marital status, education status, living alone, systolic/diastolic blood pressure, fluid intake, gravity, parity, induced abortion, breastfeeding, hormone therapy, menopause etiology, and histories of hypertension, dyslipidemia, diabetes mellitus, gout, and chronic kidney disease were recorded. BMI was calculated as body weight/height (kg/m^2^). Systolic and diastolic blood pressure measurements were also recorded. Hypertension was defined as a self-reported history of hypertension, and a systolic blood pressure of ≥ 140 mmHg and/or diastolic blood pressure of ≥ 90 mmHg. Chronic kidney disease was defined as a glomerular filtration rate lower than 60 mL/min/1.73 m^2^ estimated according to the 2021 Chronic Kidney Disease Epidemiology Collaboration creatinine equation 30.

### Sleep assessment

The participants were asked two questions to assess their sleep duration and sleep quality through self-reported questionnaires. For sleep duration, an open-ended question was asked, “How many hours did you sleep on average every day in the past month?” The participants were divided into three groups based on sleep duration per day: “less than 7 hours”, “7 hours”, and “more than 7 hours”. The cut point of 7 hours per day was based on previous research that reported the lowest risk of overall mortality in those who slept over 7 hours per night 31.

For sleep quality, the participants were asked, “How was your sleep quality in the past month?” The participants were categorized into five groups according to their answer as: “very poor”, “poor”, “normal”, “good”, and “very good”.

### Study outcome, incident PUD

The primary endpoint of the study was the self-reported development of PUD diagnosed by a physician. As mentioned above, participants with a prior history of PUD were excluded initially, and none of the participants in this study had a record of PUD at baseline. During follow-up, the participants were asked, “Have you been diagnosed with peptic ulcer disease?” The development of PUD was defined as the subject responding “Yes” to this question.

### Statistical analysis

Statistical analysis was performed using SPSS v20.0 for Windows (IBM Inc., Armonk, NY). Data were expressed as percentages or means ± standard deviations. One-way analysis of variance followed by a Bonferroni post hoc test was used to compare variables among the study groups. Survival curves for incident PUD survival were illustrated using the Kaplan-Meier method. The time to the development of incident PUD and covariates of risk factors were modeled using a multivariable Cox proportional hazards model, adjusted for age, sex, systolic and diastolic blood pressure, smoking status, alcohol status, exercise habits, marriage status, educational status, as well as a history of hypertension, diabetes, dyslipidemia, depression, drug abuse, bipolar disorder, schizophrenia, epilepsy, migraine, multiple sclerosis, dementia, chronic kidney disease, sleep duration and quality. The participants with sleep duration < 7 hours/day and very poor sleep quality group were treated as the reference group, which was at the lowest risk of incident PUD. A difference was considered significant at *p* < 0.05.

## Results

### Baseline characteristics of the three sleep duration groups

Baseline characteristics of the three sleep duration groups are presented in Table 1. A total of 22,561 participants (14,877 women and 7,684 men) without known PUD were enrolled, excluding those without complete baseline information and those with missing sleep data. The mean age was 50.77 ± 10.46 years, and the mean BMI was 24.12 ± 3.59 kg/m^2^. The participants were categorized into three groups based on sleep duration: less than 7 hours (*n* = 10,906, 48%), 7 hours (*n* = 6536, 29%), and more than 7 hours (*n* = 5119, 23%).

Compared to the < 7 hours/day group, the 7 hours and > 7 hours/day groups had a lower age and BMI.

### Association between sleep duration and incident PUD

Table 2 shows the multivariable Cox regression analysis for incident PUD according to sleep duration. Over an average 43 months of follow-up, 1325 participants (5.9% of the study population) developed PUD, including 682 (6.3%), 382 (5.8%), and 261 (5.1%) in the < 7 hours, 7 hours, and > 7 hours/day groups, respectively. After adjusting for confounders, sleep duration > 7 hours/day (*vs.* < 7 hours/day; hazard ratio [HR], 0.771; 95% confidence interval [CI], 0.668 to 0.890; *p* < 0.001) was significantly associated with incident PUD, whereas a duration of 7 hours/day was not (*p* = 0.201). Furthermore, sleep duration (per 1 hour/day; HR, 0.933; 95% CI, 0.889 to 0.979; *p* = 0.005) was also significantly associated with incident PUD.

**Figure 2:**
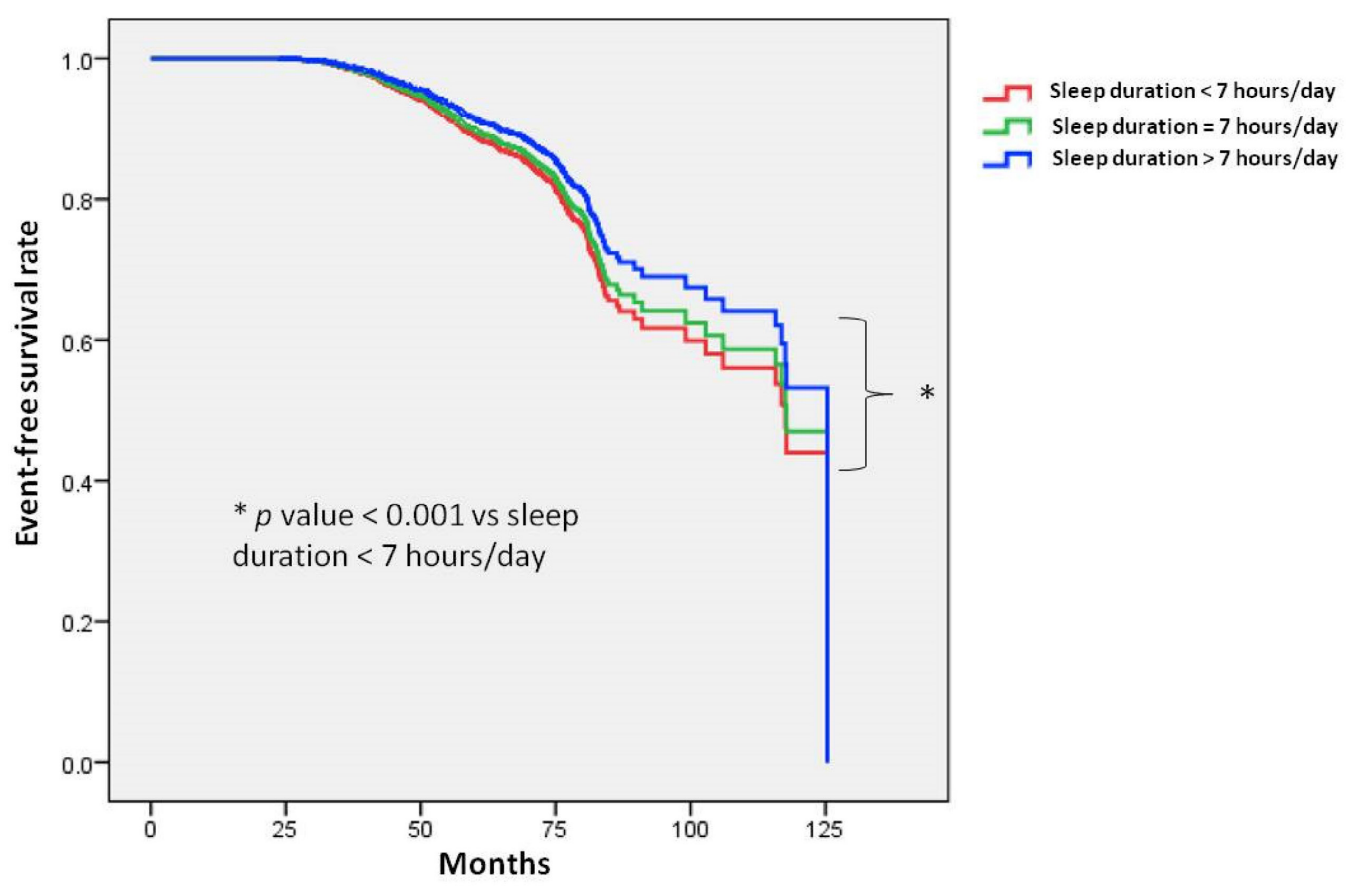
Time to incident peptic ulcer disease (PUD) was longer in participants with a sleep duration of more than 7 hours per day compared to those with less than 7 hours per day. Kaplan-Meier plot depicting the development of incident PUD based on sleep duration in 22,561 participants with follow-up data.

Figure 2 illustrates the Kaplan-Meier analysis of incident PUD among the three study groups. The time to incident PUD was longer in the > 7 hours/day group compared to the < 7 hours/day group.

### Association between sleep quality and incident PUD

Table 3 shows the multivariable Cox regression analysis for incident PUD according to sleep quality. After adjusting for confounders, the participants with poor sleep quality (*vs.* very poor quality; HR, 0.649; 95% CI, 0.491 to 0.858; *p* = 0.002), normal sleep quality (*vs.* very poor quality; HR, 0.611; 95% CI, 0.469 to 0.795; *p* < 0.001), good sleep quality (*vs.* very poor quality; HR, 0.507; 95% CI, 0.382 to 0.671; *p* < 0.001), and very good sleep quality (*vs.* very poor quality; HR, 0.493; 95% CI, 0.367 to 0.662; *p* < 0.001) were significantly associated with incident PUD. Progressively lower HRs were observed in groups with better sleep quality compared to the group with very poor sleep quality.

**Figure 3:**
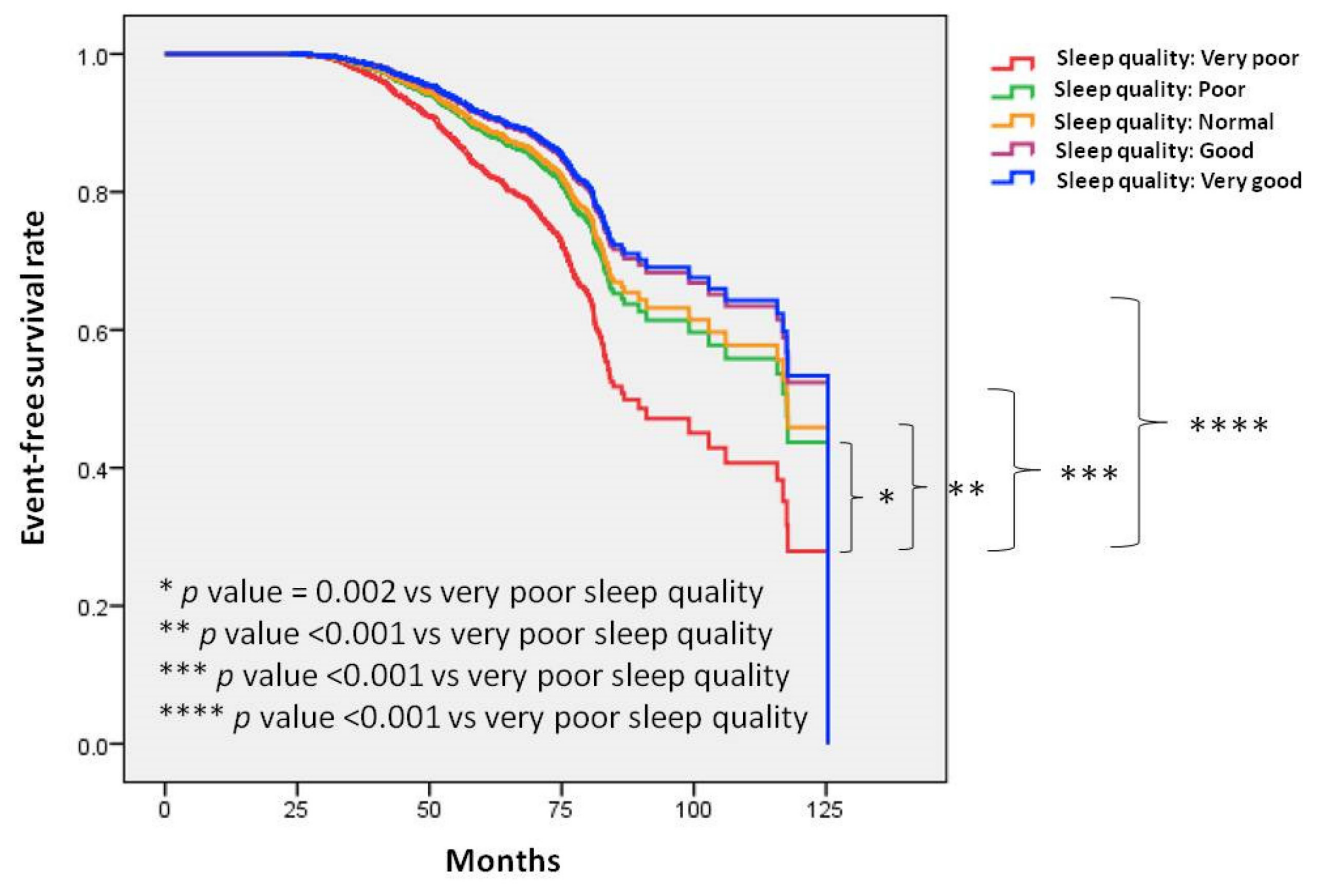
Time to incident peptic ulcer disease (PUD) was longer in participants with very good sleep quality compared to those with very poor sleep quality. Similar trends were observed when comparing good, normal, and normal sleep quality to very poor sleep quality. Kaplan-Meier plot depicting the development of incident PUD based on sleep quality in 22,561 participants with follow-up data.

Figure 3 illustrates the Kaplan-Meier analysis of incident PUD among the five sleep quality groups. The time to incident PUD was longer in the participants with very good sleep quality compared to those with very poor sleep quality. Similar trends were observed when comparing good, normal, and normal sleep quality to very poor sleep quality.

### Subgroup analysis: the association between sleep duration and incident peptic ulcer disease

**Figure 4:**
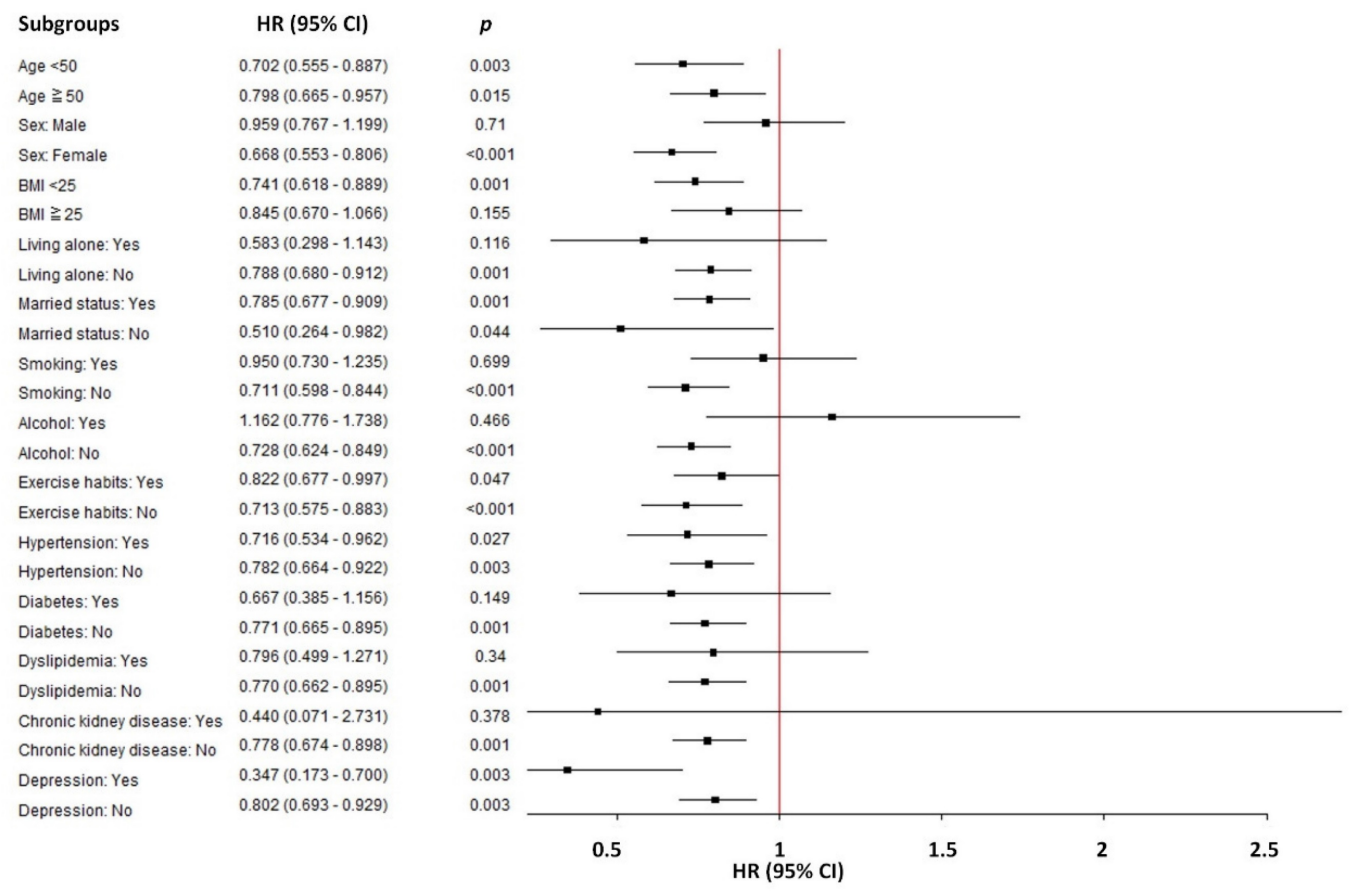
Subgroup analyses between sleep duration (sleep duration > 7 hours/day *vs.* < 7 hours /day) and the risk of incident peptic ulcer disease.

In a subgroup analysis of the association between sleep duration (sleep duration > 7 hours/day *vs.* < 7 hours /day) and the risk of incident PUD, significant variations in HR were observed across different demographics and lifestyle factors (Table 4 and Figure 4). In participants with sleep duration > 7 hours/day, female sex, BMI < 25 kg/m^2^, non-living alone, non-smokers, non-drinkers, no diabetes, no dyslipidemia, and no chronic kidney disease demonstrated a significantly reduced risk of PUD compared to sleep duration < 7 hours/day.

## Discussion

In this longitudinal study of a representative sample of the Taiwanese population, we explored associations between sleep duration and sleep quality with the development of PUD in 22,561 participants over a 43-month period. The results showed that longer sleep duration and better sleep quality were significantly associated with a lower risk of incident PUD. Moreover, for every additional hour of sleep, there was a 0.93-fold decrease in the risk of developing PUD.

The first notable aspect of this research is that a long sleep duration was associated with a low risk of incident PUD. Previous studies have reported associations between sleep duration and adverse health outcomes including cardiovascular disease, obesity, hypertension, type 2 diabetes, metabolic syndrome, immunosuppression, decline in renal function, and overall mortality 2, 8-15. Gastrointestinal diseases, including gastroesophageal reflux disease, irritable bowel syndrome, inflammatory bowel disease, colorectal cancer, and liver diseases, have also been reviewed in relation to sleep dysfunction 25, 32. However, little research has investigated the associations between sleep duration and sleep quality with the development of PUD. Ko et al. reported that women who slept less than 7 hours a night had nearly twice the risk of having PUD compared to those who slept more than 9 hours in a Korean population 29. In addition, a longer sleep duration (≥ 9 hours) tended to protect against PUD in men, but without significance 29.

Another notable finding of our research is that good sleep quality was associated with a low risk of incident PUD. Fang et al. also investigated the association between sleep quality and peptic ulcer recurrence in older patients after H. pylori eradication, and found that poor objective sleep quality, defined as longer sleep onset latency and more nighttime awakenings, increased the risk of peptic ulcer recurrence 26. Similar results were observed in another study of patients who had recovered from peptic ulcer bleeding after endoscopic or medical treatment, in whom the risk of peptic ulcer rebleeding was found to be higher in those with poorer objective sleep quality 27. In contrast, longer sleep duration and better sleep efficiency have been reported to protect against ulcer recurrence and rebleeding 27. Patients with sleep apnea have also been shown to be at risk of sleep disturbance 33. Episodes of intermittent nocturnal hypoxemia result in systemic inflammation, oxidative stress, and sympathetic activation 34. Increasing evidence has shown a link between moderate-to-severe sleep apnea and cardiovascular diseases including hypertension, coronary artery disease, atrial fibrillation, heart failure, and stroke 35. Some research has indicated an association between sleep apnea and gastroesophageal reflux disease, and its impact on PUD is still being studied 36. Shiao et al. reported a 2.4-fold higher risk of peptic ulcer bleeding in patients with sleep apnea in a study using data from the Taiwan National Health Insurance Research Database 28. Zha et al. conducted a Mendelian randomization analysis to clarify the causal relationship between sleep quality and PUD, and found that an increased incidence of insomnia was related to a higher risk of PUD, without evidence of reverse causality 37. The result indicated that insomnia was a risk factor for PUD, rather than a complication of PUD.

However, the mechanism between sleep and PUD remains unclear. Possible factors determining how sleep influences the risk of PUD include gastric mucosal blood flow, circadian rhythm, superoxide generation, the immune system, and the levels of key hormones such as melatonin and leptin 38-42. Gastric mucosal blood flow, which increases during rapid eye movement (REM) sleep, is currently considered to be the primary factor in gastric mucosal protection 39. Mucosal blood flow is regulated by systemic and local metabolic factors such as prostaglandins and leukotrienes 39. Increased mucosal blood flow supports the proper structure of the mucosa, promotes the secretion of mucus and bicarbonate ions, and consequently accelerates peptic ulcer healing 43. In an animal study, gastric mucosal epithelial cell loss and ischemic damage to the epithelium were observed in rats deprived of REM sleep 38. The resulting mucosal ischemia weakens gastric mucosal defense and repair mechanisms.

Another possible explanation is melatonin secretion. Melatonin has emerged as another pivotal factor in gastroprotection, possibly due to its stimulation of bicarbonate, antioxidative actions, and the ability to increase angiogenesis and accelerate mucosal microcirculation 44-46. Melatonin is synthesized in the pineal gland as an endocrine hormone, and is produced by intestinal enterochromaffin cells as a paracrine hormone 47. The pineal gland secretes melatonin in a circadian pattern, with the highest amounts released during the nighttime, and this may play an important role in the nocturnal control of duodenal alkaline secretion and mucosal protection 40. Melatonin also scavenges free radicals, and thereby has an anti-inflammatory effect 48, 49. The addition of oral melatonin and its precursor L-tryptophan to proton pump inhibitor therapy has been shown to significantly accelerate ulcer healing in humans 42.

Another associated key hormone, leptin, has also been shown to have gastroprotective and ulcer-healing activities, possibly due to its angiogenetic properties 50. A previous study reported that plasma leptin levels were significantly increased in patients who were treated with a regimen including melatonin for H. pylori-related peptic ulcers 41. The angiogenesis effect has been postulated to be induced by the stimulation of nitric oxide, endogenous prostaglandins, and growth factors such as transforming growth factor-alpha and vascular endothelial growth factor, leading to an increase in mucosal blood flow 50.

Another possible mechanism relates to the gut-brain axis, which involves bidirectional interactions between the central autonomic and enteric nervous systems 51. The gut-brain axis regulates intestinal immune activation, intestinal permeability, enteric reflex, and entero-endocrine signaling 40. Sleep disturbance may disrupt circadian physiology and subsequently affect the brain-gut axis. The circadian rhythm is governed by the suprachiasmatic nucleus (SCN) in the anterior hypothalamus, and it regulates the sleep-wake cycle, body temperature, blood pressure, immune response, and hormone secretion 1, 52, 53. The circadian system also influences gastric acid secretion, with variations observed during different sleep stages 54. Acid secretion decreases with deeper sleep stages, and only minimal amounts occur during REM phases, possibly resulting from decreased plasma noradrenaline and histamine release 55. Arousal or waking periods have been shown to correlate with increased acid output 56.

Trefoil factor family 2 (TFF2) protein may also play a role in the interaction between sleep and PUD. TFF2 protein is produced in the gastrointestinal mucosa, and it inhibits gastric acid secretion and affects healing of the epithelium 57. The diurnal variation of TFF2 concentration in the alimentary tract, with high levels during the night and early morning, suggests that the cytoprotective effects mainly occur during sleep 57. Sleep deprivation has been shown to disrupt the normal TFF2 rhythm and potentially predispose to increased gastric morbidity 58.

Subgroup analyses revealed that the protective effect of sleep duration was more pronounced among specific subgroups, including female sex, BMI < 25 kg/m^2^, non-living alone, non-smokers, non-drinkers, no diabetes, no dyslipidemia, and no chronic kidney disease. This sensitivity analysis reinforces our conclusion that sleep duration > 7 hours/day is significantly associated with a reduced risk of PUD. Some sub-groups are not listed in Table 4 because the number of cases is too small, such as dementia, Parkinson's disease and substance use disorder. However, it can be seen from Figure 4 that almost all sub-groups are to the left. In other words, no matter what sub-group, sleep has a tendency to reduce PUD. These findings provide valuable insights into lifestyle factors influencing PUD risk and lay the groundwork for future research in this area.

The main strengths of this study lie in its incorporation of a large community-based cohort, offering comprehensive follow-up data and detailed information. However, there are also some limitations. First, the presence of PUD was assessed using self-reports of a previous diagnosis made by a physician. Since the definite diagnosis of peptic ulcers involves direct visualization of the ulcer on endoscopy, the incidence of the disease may have been overestimated. In addition, some of the patients may have had asymptomatic ulcers and thus not been diagnosed with PUD. However, the use of self-reported diagnoses has been validated in other studies 59. Second, information on sleep duration and sleep quality was obtained through questionnaires. However, a previous study demonstrated that self-reported sleep duration was sufficient, given the concordance between self-reported sleep duration and that derived from actigraphy 60. To investigate more detailed aspects of sleep quality, including sleep latency and sleep sufficiency, further research may be conducted with information acquired through established assessment tools such as the Pittsburgh Sleep Quality Index. Third, data on H. pylori status and use of NSAIDs, steroids, chemotherapy agents, aspirin and antiplatelet therapy, which are established risk factors for PUD development, are not included in the TWB database. However, some previous studies demonstrated that sleep quality is significantly associated with PUD, even adjusted for NSAIDs use 61 and H. pylori 26.

In conclusion, longer sleep duration and good sleep quality were significantly associated with low risks of incident PUD. Furthermore, for every additional hour of sleep, there was a 0.93-fold decrease in the risk of PUD development. The effect of both sleep duration and quality on subsequent PUD development suggests the importance of optimizing the quantity and quality of sleep in the general population.

## Figures and Tables

**Table 1:** Baseline characteristics of the study subjects in the Taiwan Biobank cohort divided by sleep duration

	All	< 7 hours	7 hours	> 7 hours	
Characteristics	*n* = 22,561	*n* = 10,906	*n* = 6536	*n* = 5119	*p*
Age, years	50.77 ± 10.46	51.37 ± 10.35	50.17 ± 10.44^*^	50.24 ± 10.66^*^	< 0.001
Female, n (%)	14,877 (65.9)	7212 (66.1)	4283 (65.5)	3382 (66.1)	0.704
Body mass index, kg/m^2^	24.12 ± 3.59	24.21 ± 3.64	24.00 ± 3.46^*^	24.07 ± 3.65^*^	< 0.001
Living alone, yes, n (%)	1,435 (6.4)	734 (6.7)	367 (5.6)	334 (6.5)	0.012
Married status, ever, n (%)	20,503 (90.9)	9954 (91.3)	5935 (90.8)	4614 (90.1)	0.064
Ever smoking, yes, n (%)	5232 (23.2)	2518 (23.1)	1457 (22.3)	1257 (24.6)	0.015
Alcohol status, ever, n (%)	1810 (8.0)	832 (7.6)	527 (8.1)	451 (8.8)	0.037
Exercise habits, yes, n (%)	10,252 (45.4)	5066 (46.5)	2942 (45.0)	2244 (43.8)	0.006
Educational status, n (%)					< 0.001
Elementary school, n (%)	1656 (7.3)	907 (8.3)	359 (5.5)	390 (7.6)	
Middle to high school, n (%)	9961 (44.2)	4867 (44.6)	2834 (43.4)	2260 (44.1)	
University and above, n (%)	10,944 (48.5)	5132 (47.1)	3343 (51.1)	2469 (48.2)	
Comorbidities, n (%)					
Hypertension	4573 (20.3)	2260 (20.7)	1250 (19.1)	1063 (20.8)	0.024
Diabetes	1159 (5.1)	576 (5.3)	306 (4.7)	277 (5.4)	0.133
Dyslipidemia	1541 (6.8)	772 (7.1)	425 (6.5)	344 (6.7)	0.323
Chronic kidney disease	168 (0.7)	73 (0.7)	46 (0.7)	49 (1.0)	0.128
Depression	638 (2.8)	331 (3.0)	150 (2.3)	157 (3.1)	0.009
Substance use disorder	2 (0.0)	1 (0.0)	1 (0.0)	0 (0.0)	0.684
Bipolar disorder	104 (0.5)	50 (0.5)	31 (0.5)	23 (0.4)	0.979
Schizophrenia	33 (0.1)	11 (0.1)	5 (0.1)	17 (0.3)	< 0.001
Epilepsy	71 (0.3)	28 (0.3)	21 (0.3)	22 (0.4)	0.188
Migraine	565 (2.5)	260 (2.4)	159 (2.4)	146 (2.9)	0.190
Multiple sclerosis	6 (0.0)	5 (0.0)	1 (0.0)	0 (0.0)	0.202
Parkinson's disease	17 (0.1)	11 (0.1)	3 (0.0)	3 (0.1)	0.389
Dementia	7 (0.0)	3 (0.0)	2 (0.0)	2 (0.0)	0.927

^*^p < 0.05 compared with sleep duration < 7 hours group.

**Table 2:** Multivariate-adjusted hazard ratios for incident peptic ulcer disease according to sleep duration (*n* = 22,561)

Sleep duration	Peptic ulcer cases / subjects n, %)	HR (95% CI)	*p*
< 7 hours/day	682/10,906 (6.3)	reference	-
7 hours/day	382/6536 (5.8)	0.921 (0.812 - 1.045)	0.201
> 7 hours/day	261/5119 (5.1)	0.771 (0.668 - 0.890)	< 0.001
Per hour of sleep	-	0.933 (0.889 - 0.979)	0.005

Values expressed as hazard ratio (HR) and 95% confidence interval (CI). Covariates in the multivariable-adjusted model included sleep duration, age, sex, smoking status, alcohol status, exercise habits, marriage status, educational status, as well as the history of hypertension, diabetes, dyslipidemia, depression, drug abuse, bipolar disorder, schizophrenia, epilepsy, migraine, multiple sclerosis, dementia, and chronic kidney disease.

**Table 3:** Multivariate-adjusted hazard ratios for incident peptic ulcer disease according to sleep quality (*n* = 22,561)

Sleep quality	Peptic ulcer cases / subjects (n, %)	Adjusted HR (95% CI)	*p*
Very poor	62/570 (10.9)	reference	-
Poor	247/3969 (6.2)	0.649 (0.491 - 0.858)	0.002
Normal	601/9932 (6.1)	0.611 (0.469 - 0.795)	< 0.001
Good	247/4872 (5.1)	0.507 (0.382 - 0.671)	< 0.001
Very good	168/3218 (5.2)	0.493 (0.367 - 0.662)	< 0.001

Values expressed as hazard ratio (HR) and 95% confidence interval (CI). Covariates in the multivariable-adjusted model included sleep quality, age, sex, smoking status, alcohol status, exercise habits, marriage status, educational status, as well as the history of hypertension, diabetes, dyslipidemia, depression, drug abuse, bipolar disorder, schizophrenia, epilepsy, migraine, multiple sclerosis, dementia, and chronic kidney disease.

**Table 4:** Subgroup analysis: the association between sleep duration and incident peptic ulcer disease

Subgroup	Sleep duration > 7 hours/day *vs.* < 7 hours /day HR (95% CI)	*p*
Age		
< 50 years	0.702 (0.555 - 0.887)	0.003
≥ 50 years	0.798 (0.665 - 0.957)	0.015
Sex		
Male	0.959 (0.767 - 1.199)	0.710
Female	0.668 (0.553 - 0.806)	<0.001
Body mass index		
< 25 kg/m^2^	0.741 (0.618 - 0.889)	0.001
≥ 25 kg/m^2^	0.845 (0.670 - 1.066)	0.155
Living alone		
Yes	0.583 (0.298 - 1.143)	0.116
No	0.788 (0.680 - 0.912)	0.001
Married status		
Yes	0.785 (0.677 - 0.909)	0.001
No	0.510 (0.264 - 0.982)	0.044
Smoking		
Yes	0.950 (0.730 - 1.235)	0.699
No	0.711 (0.598 - 0.844)	<0.001
Alcohol		
Yes	1.162 (0.776 - 1.738)	0.466
No	0.728 (0.624 - 0.849)	<0.001
Exercise habits		
Yes	0.822 (0.677 - 0.997)	0.047
No	0.713 (0.575 - 0.883)	<0.001
Hypertension		
Yes	0.716 (0.534 - 0.962)	0.027
No	0.782 (0.664 - 0.922)	0.003
Diabetes		
Yes	0.667 (0.385 - 1.156)	0.149
No	0.771 (0.665 - 0.895)	0.001
Dyslipidemia		
Yes	0.796 (0.499 - 1.271)	0.340
No	0.770 (0.662 - 0.895)	0.001
Chronic kidney disease		
Yes	0.440 (0.071 - 2.731)	0.378
No	0.778 (0.674 - 0.898)	0.001
Depression		
Yes	0.347 (0.173 - 0.700)	0.003
No	0.802 (0.693 - 0.929)	0.003

Values expressed as hazard ratio (HR) and 95% confidence interval (CI).
